# Editorial: Epigenetic and Transcriptional Dysregulations in Cancer and Therapeutic Opportunities

**DOI:** 10.3389/fgene.2022.857380

**Published:** 2022-02-22

**Authors:** Rais A. Ansari, Ata Abbas

**Affiliations:** ^1^ Department of Pharmaceutical Sciences, Nova Southeastern University, Fort Lauderdale, FL, United States; ^2^ Division of Hematology and Oncology, Department of Medicine, Case Western Reserve University, Cleveland, OH, United States; ^3^ Case Comprehensive Cancer Center, Case Western Reserve University School of Medicine, Cleveland, OH, United States

**Keywords:** epigenetics, transcription, transcriptional dysregultaion, cancer, cancer therapies

**FIGURE 1:**
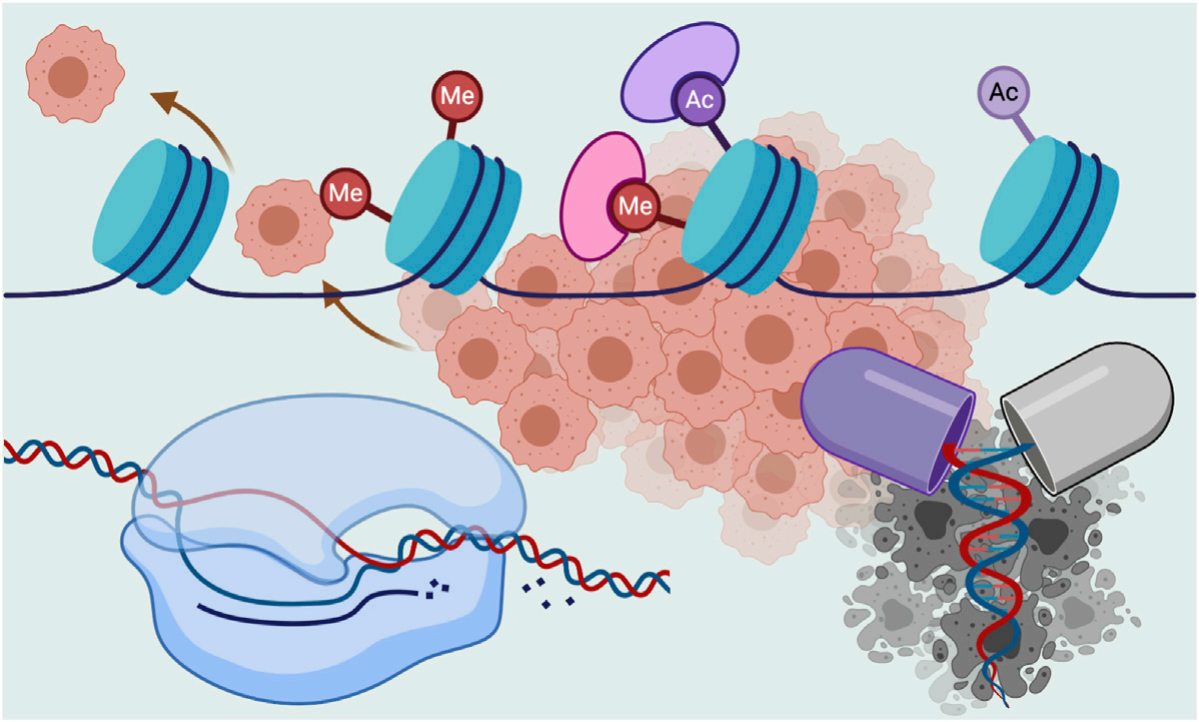
Alteration of epigenetic and transcriptional processes often contribute in carcinogenic events. These alterations can also serve as novel therapeutic targets and biomarkers (created with BioRender.com).

Aberrations in epigenetic regulation at various levels, including DNA methylation, chromatin architecture, and regulatory RNAs, are often associated with, and significantly contribute in most carcinogenesis (Jones and Baylin, 2007; Baylin and Jones, 2016). Transcriptional dysregulation is another hallmark of nearly all kinds of cancers (Hanahan, 2022). Gene transcription is a complex process and highly regulated at various stages as well as at the post-transcriptional level (Corbett, 2018; Roeder, 2019). Epigenetic and transcriptional events often influence each other, e.g., DNA methylation and histone modifications regulate gene transcription, and transcriptional processes can modify chromatin architectures (Bonasio et al., 2010; Gibney and Nolan, 2010). Increasing numbers of evidence suggest epigenetic and transcriptional dysregulations play vital roles in carcinogenesis, including metastasis, aggressiveness, and recurrence of malignancies (Bradner et al., 2017; Hanahan, 2022). An in-depth understanding of both epigenetic and transcriptional processes and alterations in their regulations are needed to better understand tumor pathobiology and to improve clinical management (Figure 1). Epigenetic and transcriptional dysregulations also confer therapeutic vulnerabilities and remarkably, offer novel biomarkers and therapeutic targets (Gonda and Ramsay, 2015; Cheng et al., 2019; Lu et al., 2020; Vervoort et al., 2021).

Genome-wide transcriptional control is often dysregulated in cancer. In this research topic, Parrello et al. discussed the possibility of targeting factors that control global transcriptional regulation. Li et al. discussed the dual roles of CBX7, a component of polycomb repressive complex, in cancer where it can either help in cancer progression by downregulating tumor suppressor genes or help cancer suppression by modulating cell cycle related proteins. CBX7 interacts with various regulatory RNAs, including micro RNAs, long non-coding RNAs, circular RNAs. Regulatory RNAs play a significant role in carcinogenesis, including chemo-resistance (Lan et al., Gareev et al., and Zhang et al.). Sun et al. showed that plasma-derived exosomal micro RNA, miR-2276-5p in glioma patients could serve as a potential diagnostic and prognostic marker. Transcriptomics based gene signatures are emerging as promising biomarkers in cancer (Jin et al. and Fang et al.).

Epigenetic landscape is altered in cancer cells that results in transcriptional dysregulation. Various dietary components have ability to modulate epigenetic aberration (Fatima et al. and Raina et al.). Abbas et al. reported that maternal diet rich in omega-3 fatty acid can reprogram epigenetic and transcriptomic landscapes in F1 generation mice and provide resistance to breast cancer development. Pu et al. reported that methylation profiles of zinc finger genes, specially *ESR1* and *ZNF132*, could be potential biomarkers for the early diagnosis of colorectal cancer patients carrying KRAS mutations. Another study by Gua et al. showed that *APOA1* gene is downregulated by DNA methylation in hepatocellular carcinoma that could be a potential biomarker to predict survival. Role of NTPCR in epithelial ovarian cancer (Shang et al.) and FGFR1–GLI1 axis as a potential therapeutic target in breast cancer (Riaz et al.) were also reported.

Gazova et al. used CRISPR-Cas9 to generate homozygous inactivating mutation in *USP16* gene using leukemia cell line and studied how these cells adapt to the extreme selection pressure through compensatory pathways. Authors also cautioned targeting USP16 in leukemia as cancer could develop resistant to USP16 inhibitors. A timely review by Amir et al. discussed the usefulness of combination therapy of tyrosine kinase inhibitors with epigenetic drugs in chronic myeloid leukemia. Leszczynska et al. reviewed the emerging therapeutic approaches against pediatric high-grade gliomas, particularly those having mutations in genes coding for histone 3 variants that result in substitution of lysine at 27 to methionine.

In conclusion, epigenetic aberration and transcriptional homeostasis disruptions are associated with cancer. In-depth understanding of these processes and their interdependencies is needed to better understand carcinogenesis and to develop novel and effective therapeutic approaches.
